# The Yield Prediction of Synthetic Fuel Production from Pyrolysis of Plastic Waste by Levenberg–Marquardt Approach in Feedforward Neural Networks Model

**DOI:** 10.3390/polym11111853

**Published:** 2019-11-10

**Authors:** Faisal Abnisa, Shafferina Dayana Anuar Sharuddin, Mohd Fauzi bin Zanil, Wan Mohd Ashri Wan Daud, Teuku Meurah Indra Mahlia

**Affiliations:** 1 Department of Chemical and Materials Engineering, Faculty of Engineering, King Abdulaziz University, Jeddah 21589, Saudi Arabia; 2 Department of Chemical Engineering, Faculty of Engineering, University of Malaya, Kuala Lumpur 50603, Malaysia; dayanareen@gmail.com (S.D.A.S.); ashri@um.edu.my (W.M.A.W.D.); 3 Department of Chemical Engineering, Faculty of Engineering, Technology & Built Environment, UCSI University, Cheras 56000, Malaysia or; 4 School of lnformation, Systems and Modelling, Faculty of Engineering and Information Technology, University of Technology Sydney, Sydney, NSW 2007, Australia; TMIndra.Mahlia@uts.edu.au

**Keywords:** plastic waste, pyrolysis, artificial neural network, prediction, fuel

## Abstract

The conversion of plastic waste into fuel by pyrolysis has been recognized as a potential strategy for commercialization. The amount of plastic waste is basically different for each country which normally refers to non-recycled plastics data; consequently, the production target will also be different. This study attempted to build a model to predict fuel production from different non-recycled plastics data. The predictive model was developed via Levenberg-Marquardt approach in feed-forward neural networks model. The optimal number of hidden neurons was selected based on the lowest total of the mean square error. The proposed model was evaluated using the statistical analysis and graphical presentation for its accuracy and reliability. The results showed that the model was capable to predict product yields from pyrolysis of non-recycled plastics with high accuracy and the output values were strongly correlated with the values in literature.

## 1. Introduction

The depletion of fossil fuels and the impact of their applications on the environment have forced many scientists around the world to innovate in generating alternative energy [1]. Some countries have successfully implemented wind and solar energy [2], some are more toward bioenergy [3], and the others are trying to produce biodiesel [4] through finding new alternative sources [5] or fuel blends strategy [6,7,8]. Although solar and wind energies are environmentally friendly technologies, they actually depend on the environment condition, and as a consequence, the new problem on how to eliminate the dependency on these natural sources has emerged. This issue then has inspired the researcher to develop the energy storage device by searching the reliable materials which have excellent thermal properties and are stable [9,10,11]. This scenario showed that a simple idea of developing alternative energy still needs to be explored further to encourage creating the future of energy security.

Through extensive research and technology development, plastic waste conversion to fuels begins to gain traction in energy research. Plastics are made from the petroleum-based material which basically having a high calorific value, thus their conversion to fuels is highly possible. The strategy to pyrolyze plastic waste to be liquid fuel has been proven by many researchers as one of the best techniques to produce alternative energy in regard to overcoming the depletion of conventional liquid fuel. The current research findings have proven that the pyrolysis of plastic waste managed to produce high yield of liquid fuel more than 80 wt % [12]. The properties of the pyrolysis liquid were also very close to the conventional diesel. Besides, the oil also had better features compared to the oil from wood biomass such as having a high calorific value around 40 MJ/kg, low water content, and low amount of oxygen [12,13,14,15]. Moreover, the low sulfur content in comparison to the standard diesel has made it more environmentally friendly to be used as fuel [16]. 

Because of its properties and availability, plastic waste has become one of the most potential sources that can be globally commercialized for pyrolysis oil production. Prior to commercialization, some of the important aspects that need to be considered are the quantity of feedstock and production target [17]. Basically, plastic waste can be obtained from municipal solid waste and is comprised of different types of plastics which are high-density polyethylene (HDPE), low-density polyethylene (LDPE), polypropylene (PP) and polystyrene (PS), polyethylene terephthalate [18] and polyvinyl chloride (PVC). To obtain accurate information about plastic waste quantity, the data of non-recycled plastics (NRP) waste provided by the waste management authorities can be used as a reference. By definition, NRP is the plastic waste which accumulates in municipal solid waste (MSW) or in materials recovery facility residue which is not redirected for the recycling process [19]. The use of NRP data in the pyrolysis process will contribute to producing more convincing data yield which then can be used to calculate the real target production of liquid fuel, as well as the quality standard can also be determined [20]. 

The amount of NRP is different in every country based on the production and application of plastics in daily life. Consequently, the oil production target will also be varied depending on the accumulation of these wastes, where the ratio of each plastic waste plays an important role. Because of the varieties of plastic ratios, many data are needed for analysis; as a result, many experimental works require to be performed for preliminary designs. Many different sets of preliminary design studies actually are not really desired because they are not only time consuming but also decrease the productivity in research area and need high expenses to support the research. In order to overcome this problem, a model which has an ability to predict the product yield from pyrolysis of different composition of plastic waste needs to be developed. 

Developing a model to predict the oil quantity from pyrolysis of plastic waste seems less complex than the pyrolysis of wood biomass. Plastic is a synthetic material in which the main compositions for each type is usually similar, while wood biomass is basically influenced by the nature of the non-homogeneous lignocellulosic compound. In terms of the reaction temperature, all type of plastics has a uniform trend in completing the decomposition. According to Lopez et al. [21] and Papuga et al. [18], all of plastic waste completely pyrolyze basically at 500 °C.

There are many advanced techniques to analyze the experimental procedure and their results such as extreme learning machine, response surface methodology, ant colony, and artificial neural network (ANN) [22,23,24,25,26]. However, ANN is one effective tool to develop a predictive model. ANNs have the ability to capture the complex non-linear relationship between dependent and independent variables in a system [27]. Other advantages of using ANNs are less statistical training, do not require any mathematical correlation between the interactions of variables, and have the ability to allow the model to be updated themselves [28]. The high flexibility of the ANNs has made them widely used in various applications as one of the most reliable and predictive tools. ANNs have been used extensively in predicting the HHV of different types of biomass [29], prediction of sugar yields during hydrolysis of lignocellulosic biomass [30], prediction of damaged caused by random fatigue loading that occurred in many structural and machine components [31], prediction of daily watershed runoff [32], and also prediction of kinetic parameters of biomass pyrolysis from its constituents [33]. 

Antwi et al. [34] studied the comparison between ANN and multiple nonlinear regressions to estimate the biogas and methane yield in an up-flow anaerobic sludge blanket reactor treating potato sludge processing wastewater. Among these two models, they found that ANN with backpropagation algorithm demonstrated significant performance in estimating the yield of biogas and methane in anaerobic reaction. Rostami et al. [35] investigated the performance of the ANN model versus two commonly used isotherm models (Sips and Langmuir) to estimate CO_2_ adsorption on activated carbon. They discovered that the ANN-based algorithm showed much better accuracy and efficiency than the isotherm models. Besides, Chen et al. [36] also concluded that ANN model was better than the K-nearest neighbors mode (a linear method) since ANN produced higher pattern recognition rate for the identification of tea grade level. Hence, the significant performances of ANNs in giving accurate prediction than other models in literature have been proven in many areas and their reliabilities are undoubted. 

This study aims at predicting the potential product yields from the pyrolysis of plastic waste that consists of different composition of plastic ratios by developing a predictive neural network model. The study was started by collecting the data from experimental works and literature for training and validation purposes. An empirical-based model was then developed based on the training data set via Levenberg-Marquardt [22] feed-forward neural network (FANN) while the validation data were used to test the predictive model. In addition, relevant discussions regarding the modeling results were also included. 

## 2. Materials and Methods

### 2.1. Materials

In pyrolysis of NRP, all consumable plastics such as HDPE, LDPE, PP, and PS were included as feedstock in the process while PET and PVC were excluded because of their harmful properties. Pyrolysis of PET is not recommended in pyrolysis since the degradation of this plastic produces a harmful product such as benzoic acid which may disturb the performance of process equipment. Benzoic acid is a general sublime that could clog the piping and heat exchanger, thus need serious attention if running at industrial scale. Pyrolysis of PVC releases hydrogen chloride which deteriorates the fuel quality and causes damage to the equipment. Besides, the PVC waste accumulation in MSW is very minimal, about less than 3% in the plastic waste category which is very limited and thus omitted in this experiment. The source of plastic waste was from the post-consumer polymer waste stream in Malaysia. To remove the impurities, the collected plastics were washed with water and then dried using the oven at 105 °C for 24 h. The dried plastic waste was ground into small pieces using a plastic scrap grinder machine and subsequently fed into the reactor as feedstock. The plastic ratio was prepared and mixed according to the NRP data obtained from Malaysia, United States, United Kingdom, and global as shown in Table 1. 

### 2.2. Experimental Setup and Operation

**Figure 1:**
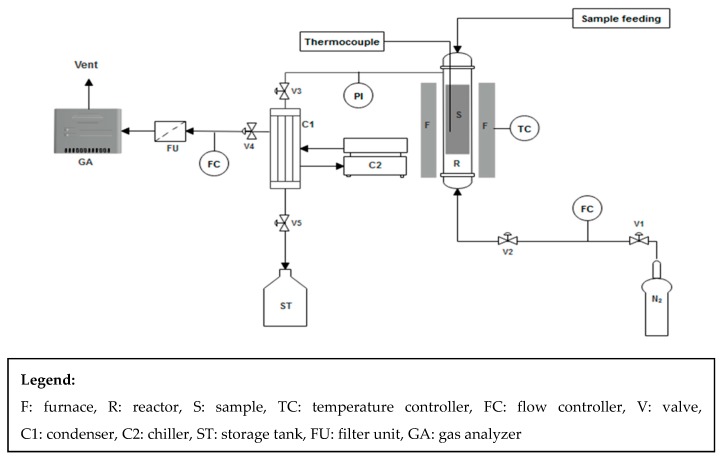
Schematic diagram of experimental setup for pyrolysis of plastic mixtures.

A stainless steel fixed-bed reactor was used for pyrolysis experiment with an internal diameter of 5.0 cm and a total length of 127 cm. A K-type thermocouple was equipped inside the reactor to monitor the temperature that is heated by an external furnace. A condenser that sustained the furnace temperature at the desired level of ±0.5 °C was connected in order to obtain the liquid by condensing the condensable products which release from the reactor. The experimental setup is illustrated in Figure 1.

About 400 g of plastic waste was prepared as a basis weight for the determination of each NRP based on the percentage shown in Table 1, excluded the PET and PVC. The pyrolysis process was performed at the reaction temperature of 500 °C with a heating rate of 20 °C/min by using the electric furnace. The reaction time was set at 30 min for each experiment. Nitrogen was selected as the carrier gas with a flow rate of 200 mL/min which helps to remove the vapors from the reaction site and minimize the secondary reaction that would reduce the yield of liquid oil.

### 2.3. Data Collection

In pyrolysis of plastics, basically, there are four types of scenarios: pyrolysis of individual plastic, pyrolysis of the plastic mixture at different ratios, co-pyrolysis of plastics with other biomass, and also catalytic pyrolysis of plastics. In developing this predictive model, pyrolysis of plastic mixture was chosen since this scenario can be considered as a representative of the NRP condition. For the purpose of oil production, there are three basic steps required for the pyrolysis process: preparation of samples, pyrolysis, and condensation. In terms of sample preparation, the selection of plastic waste is also important in ensuring good quality of liquid produced. For that reason, only four types of consumable plastics particularly HDPE, LDPE, PP, and PS were selected as input for the model development. 

Temperature is the main operating parameter in the pyrolysis process, thus it really needs to be considered for developing this model. The data collected from the literature were selected within the temperature range of 400 to 500 °C with the assumption that the processes were completed in 30 min reaction time. Particle size was assumed less than 2–3 mm based on the suggestion by Bridgewater where it was required to achieve high biomass heating rate [41]. An inert gas such as nitrogen was normally used in the pyrolysis of plastics to accelerate sweeping vapor from the hot zone (pyrolysis zone) to the cool zone (condenser). This model was developed with the assumption that the short hot vapor residence time required less than 2 s in regard to minimizing the secondary reaction and maximize oil production [42]. For the condensation stage, this model also assumed a constant temperature of 5 °C for condensation process. 

**Figure 2:**
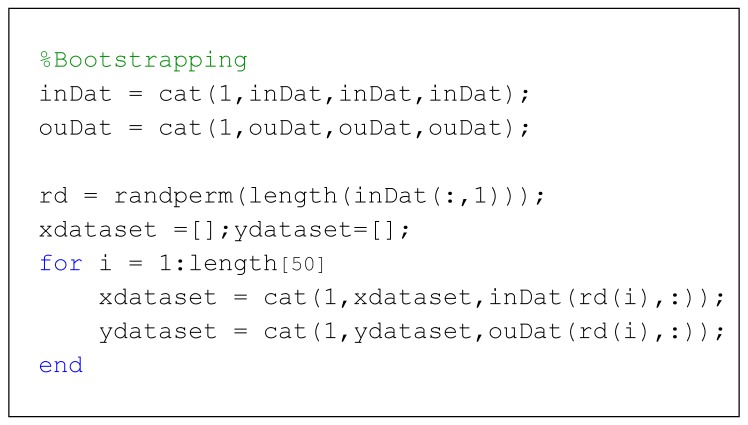
Coding used for bootstrapping the initial dataset.

Table 2 presents the plastic composition (input) and pyrolysis products (output) data obtained from the experimental works as well as from the literature that were used for FANN model development. The statistical parameters for the utilized input and output data are summarized in Table 3. The model prediction capability was only valid within the boundaries outlined in Table 3. Since only a small data set was available for FANN development, a pseudo-random bootstrapping method was applied to increase the prediction accuracy. The data set was bootstrapped ten times. The randomly sampled bootstrap set then formed the data used to train the FANN, while the unused data indicated by ‘*’ in Table 2 served to validate the FANN model. The coding for bootstrapping is presented in Figure 2. In the event of a small (limited) data set, this study limits the validation size to 10% from the total of samples [43]. This will give the FANN model enough data to have maximum the exposure to unique information on plastic composition. For this reason, the cross-validation technique is performed to reduce variability where each sample is selected at different partitions in the plastic composition.

### 2.4. Development of FANN Training Algorithm and LM Optimization Process

**Figure 3:**
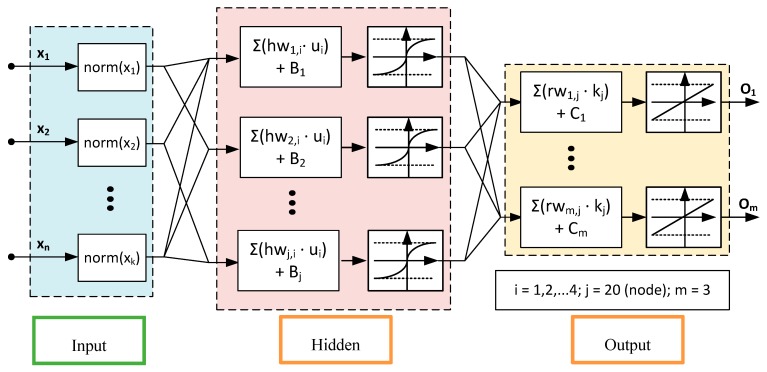
Schematic multi input multi output framework of a FANN model structure to predict the synthetic fuel production from pyrolysis of NRP.

In this study, FANN modeling was performed using the Neural Network Toolbox in Matlab. The development of the FANN model to predict the synthetic fuel production from pyrolysis of NRP used a feed-forward approach which consisted of the input layer, hidden layer, and output layer as illustrated in Figure 3. They comprised of vast number of interconnected artificial neurons or nodes. The number of neurons in input layer was the same as the number of input parameters, whereas the number of neurons in the output layer was equal to the number of dependable variables. For the present investigation, there were four neurons in the input layer which were HDPE, LDPE, PP, and PS and three neurons in the output layer corresponded to liquid, gas, and tar. As for the hidden layer, the number of neurons was set to default to ten neurons. In order to get the optimal number of hidden neurons, the trial and error approach was used to determine the number of neurons in the hidden layer to minimize the mean square error (MSE) of the model. 

Training procedure of FANN was based on the LM backpropagation algorithm. The input layer was introduced to the hidden layer by weights and biases which provided the additional parameters. The hidden layer calculated the sum of all the weighted inputs and the associated biases. Subsequently, the sum of weights from the hidden layer including the bias was passed to the neurons in output layer via sigmoidal transfer function. Finally, the output values, *y_m_* of the FANN were calculated from input variables (*x_i_*) by Equation (1).

(1) $$y_{m}=purelin\left(\overset{neuron}{\underset{j=1}{\sum }}{\left[rw_{m,j}\left(tansig\left(\overset{4}{\underset{i=1}{\sum }}{\left[hw_{j,i}\cdot x_{i}\right]}_{m}+B_{j}\right)\right)\right]}_{m}+C_{m}\right);\;m=1,2,3$$

where $hw_{j,i}$ represents the weight connecting *i*th neuron in the input layer to the *j*th neuron in the hidden layer, $B_{j}$ is the bias of the *j*th neuron in hidden layer, $rw_{m,j}$ is the weight connecting the *j*th neuron in hidden layer to the *m*th neuron in output layer, and $C_{m}$ represents the bias of the *m*th neuron in the output layer.

In this study, the FANN was developed based on the combination of a hyperbolic tangent sigmoid function (tansig) (Equation (2)) and a linear transfer function (purelin) (Equation (3)) which were used in the hidden layer and output layer respectively. The combination of these two equations managed to obtain the lowest mean square error (MSE) in various cases [34,55]

(2) $$tansig\left(u\right)=\frac{2}{1+\exp\left(-2u\right)-1}$$

(3) $$purelin\left(u\right)=u;\;u_{min}<u<u_{max}$$

**Figure 4:**
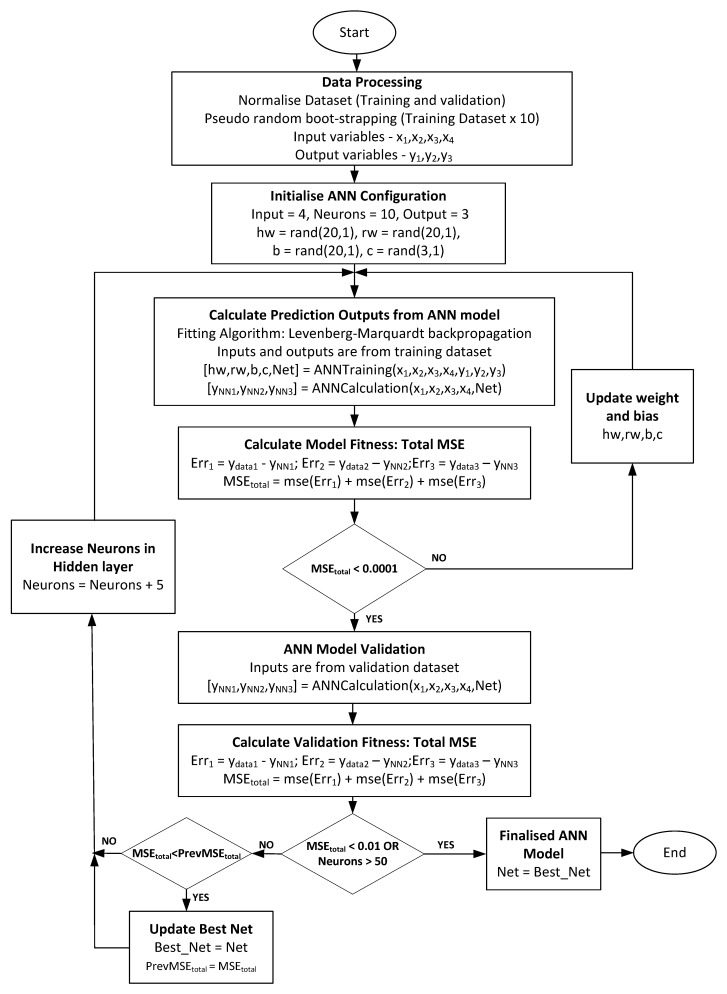
FANN model development flowchart.

The process flow of the model development is presented in Figure 4 as an overall design procedure. The dataset was prepared (as discussed in the previous section) and is being utilized in the development of FANN empirical model. Several parameters were initialized and would be optimized for many iterations to meet the model prediction fitness in either training or validation phase. The objective of the optimal solution was to achieve the best fitness of MSE between output (from the dataset, product yield) and FANN prediction. In general, there is a trade-off between accuracy in the training error and validation error and it is a common problem in the FANN system identification. In this study, the early stopping method was applied since there was a tendency of the FANN model to over-fit or under-fit the data. Thus, training could be stopped at the point of the smallest error with respect to the validation data in order to improve the generalization performance of the FANN. The training set was used as the first subset to compute the gradient in Levenberg-Marquardt regression and update the network weights and biases. The validation set was the second subset and the error obtained in this set was constantly monitored during the training process. 

## 3. Results and Discussion

### 3.1. FANN Model Selection

**Figure 5:**
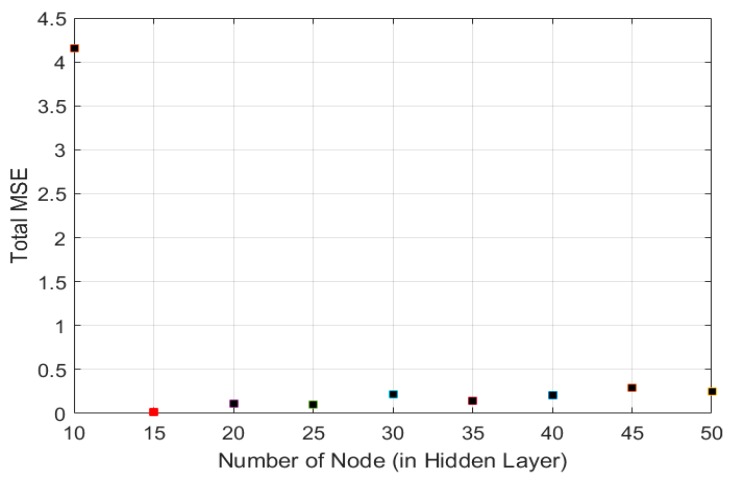
Selection of the number of nodes or neurons in the hidden layer for the ANN determination of the liquid, gas and tar.

Besides defining the number of neurons in the input and output layer, deciding the optimal number of neurons in the hidden layer is also important to determine the performance of the FANN model in minimizing the MSE. The lower value of MSE indicates better suitability of the model. Over-fitting of the model may occur if the number of neurons in the hidden layer is greater than the optimized value. When over-fitting occurs, the network tends to memorize the training pattern instead of learning to generalize the new situation. In contrast, the lesser number of neurons than the optimal value may lead to under-fitting of the model which requires more training time [56,57]. Figure 5 displays the effect of different number of neurons in a hidden layer on the MSE for outputs. From Figure 5, it can be seen that the lowest total of MSE was achieved when the number of nodes or neurons in the hidden layer turned to 15. Therefore, 15 neurons were selected in the hidden layer for the present study to avoid the over-fitting or under-fitting of model. However, to avoid high computational load, the maximum number of neuron in hidden layer was limited to 105.

**Figure 6:**
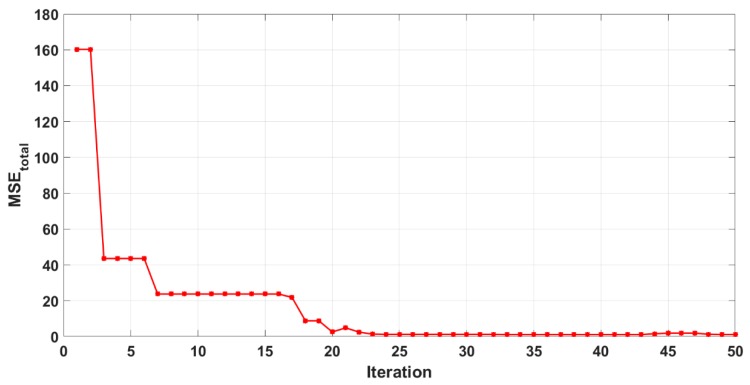
Number of training iteration for the ANN.

The training process was considered done (optimized) when the value of MSE maintained constant over several iterations. Referring to Figure 6, the weights and biases were continuously updating until the total value of MSE less than 1 with 25 iterations. 

The optimized parameters (Best Net as in Figure 4) are: the weights of neurons in the hidden layer ($hw_{j,i}$), weight of neurons in the output layer ($rw_{m,j}$), bias of inputs ($B_{j}$), and bias of outputs ($C_{m}$) are shown in Table 4. By using these parameters in the FANN model, the output values of liquid, gas, and tar can be obtained for different composition of plastics (HDPE, LDPE, PP, and PS) in pyrolysis. 

### 3.2. Evaluation of the Selected FANN Model

**Figure 7:**
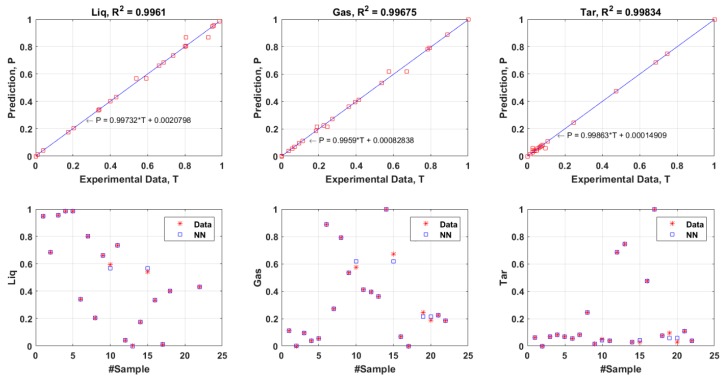
Comparison of the experimental results (from training dataset) and predicted results derived from the ANN model, $MSE_{total}=2.6419\times 10^{-4}$.

**Figure 8:**
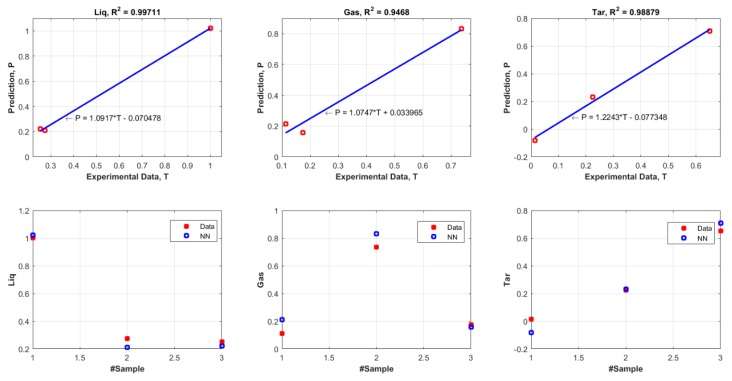
Comparisons between the experimental results (from validation dataset) and the predicted curve (ANN model), $MSE_{total}=0.0869$.

The determination of coefficient (R^2^) is a statistical measure of how well the suggested FANN model precisely reproduces the output values. A greater value of R^2^ closer to unity indicates the high reliability of the model in giving a consistent prediction. The total MSE and R^2^ values of training and validation dataset for the products of liquid, gas, and tar are shown in Figure 7 and Figure 8 respectively. From these two plots (Figure 7 and Figure 8), it can be seen that the R^2^ values for each product showed a good agreement between the experimental values and predicted values (R^2^ = above 0.9). Moreover, the values of total MSE from training dataset and validation dataset were considered low which were $2.6419\times 10^{-4}$ and 0.0869 respectively. The difference of the total MSE for both training and validation dataset was very minimal and this signified that the selected number of neurons (15 neurons) in the hidden layer was at the optimal value. These results confirmed the capability of the proposed FANN model in predicting the liquid, gas, and tar products from pyrolysis of four different kinds of plastics. 

**Figure 9:**
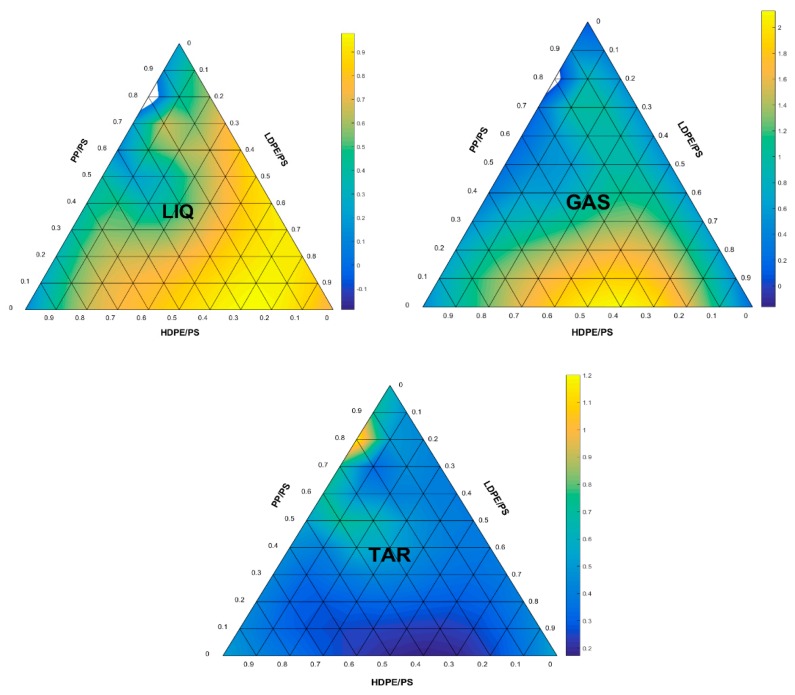
Prediction of liquid, gas, and tar yield based on the plastic composition.

Figure 9 shows the product yield based on normalized input and output data. The values are obtained based on the proposed neural network model and the normalized compositions of the plastics. The normalized compositions of plastics are relative to PS since the majority of PS had the lowest amount of composition in the dataset. The contour colors show a prediction value for every respective product for each plastic mixture. Based on the ternary plot, high liquid product can be obtained when the mixture contains a high composition of LDPE and PS which is represented by the lightest yellow area. According to Sharuddin et al. [12], the ascending order of the pyrolysis oil yield of the four thermoplastics can be sorted as HDPE, PP, LDPE, and PS. Lee et al. [58] and Onwudili et al. [59] found the same trends during pyrolysis of individual plastics with PS. Therefore, the prediction trend for liquid presented in the ternary plot agrees with this theory. For gas prediction, high amount of gas is produced when the mixture consists of HDPE/PS and LDPE/PS dominantly. This is proven in a study performed by Demirbas [60] who found that polyethylene (combination of HDPE and LDPE) produced more gas than PP during pyrolysis, while PS produced the least amount of gas. 

On the other hand, the mixture of plastics are predicted to produce very less tar in comparison to liquid and gas for all combination of plastics. Demirbas [60] and Lopez et al. [21] obtained only small amount of tar in pyrolysis of the plastic mixture which was about 1.4% and 5.5% respectively. Overall, the proposed FANN model displayed a reliable prediction of the product trends which tally with the literature. However, it should be noted that other parameters such as heating rate, temperature, and residence time may also influence the amount of tar formed in pyrolysis, as well as the amount of liquid and gas. For instance, slow heating rate at very low temperature and long residence time maximize the tar formation in pyrolysis process. Higher temperature more than 500 °C would favor the formation of gas and tar, whereas lower temperature in the range of 300–500 °C would produce more liquid product in pyrolysis of plastics [12].

## 4. Conclusions

Product yields from pyrolysis of NRP could be easily and accurately predicted with the establishment of this predictive model. The regression analysis showed that the obtained R^2^ values for both training and validation dataset were above 0.9. Besides, the total MSE values of training and validation dataset were also considered small which were $2.6419\times 10^{-4}$ and 0.1114 respectively. The prediction of liquid, gas, and tar obtained from the proposed FANN which were represented by the ternary plot also showed a strong correlation with the literature, indicating high reliability and adequacy of the model. 

## Figures and Tables

**Table 1:** Global composition of non-recycled plastics (NRP) waste consisted in municipal solid waste (MSW) [37,38,39,40].

Plastic Material	Malaysia Plastic Waste (%)	US Plastic Waste (%)	UK Plastic Waste (%)	Global Plastic Wastes (%)
PET	16.2	12.4	15.3	15.43
HDPE	26.2	17.8	13.5	16.97
PVC	3.9	5.5	3.5	3.08
LDPE	31.1	19.6	25	33.95
PP	8.2	13.9	22.2	15.43
PS	13	8.7	4	12.35

**Table 2:** Input and output data for artificial neural network (ANN) model development.

No.	Plastic Composition, Inputs (wt %)				Products, Outputs (wt %)			References
	HDPE	LDPE	PP	PS	Liquid	Gas	Tar	
1	33	22	33	11	72	na	na	[44]
2	34	34	16	16	93	6	1	[45]
3 *	8	8	68	16	90	5	5	[45]
4	8	8	16	68	92	2	6	[45]
5	16.5	16.5	33	33	92	3	5	[45]
6	22.9	45.8	9.5	9.5	70.5	28.4	1.2	[46]
7	12.5	12.5	25	50	49	47.1	3.9	[47]
8	10	10	20	40	40	42	18	[47]
9	34.57	34.58	9.57	9.57	65.94	30.47	3.59	[48]
10	32	32	32	2.5	75.4	21.9	2.7	[48]
11	25	50	25	0	79.7	14.4	5.9	[49]
12	26.2	31.1	8.2	13	29	20.89	50.11	Experimental value (Malaysia)
13	17.8	19.6	13.9	8.7	26.33	19.17	54.5	Experimental value (US)
14	13.5	25	22.2	4	44.62	39	16.38	Experimental value (UK)
15 *	17	34	15.4	12.4	43.2	9.28	47.52	Experimental value (Global)
16 *	34.57	34.58	9.57	9.57	62.35	35.53	2.12	[48]
17	44.4	0	21.2	13.3	48.7	3.7	34.6	[50]
18	29.55	29.55	25	7.2	27	0	73	[51]
19	39.5	0	34.17	16.26	53	41.5	5.5	[21]
20	24.3	24.3	24.3	26	38	53	2	[22]
21	30	30	13	18	80	13	7	[52]
22	30	30	13	18	88	10	2	[52]
23	29.4	29.4	26.9	8.7	80	12	8	[53]
24	31.25	31.25	7.29	13.5	55.07	9.79	2.82	[54]

* indicate the particular row has been excluded from the training dataset and are used for validation.

**Table 3:** The statistical parameters of the input and output data.

Statistic Parameters	Plastic Composition (wt %)				Products (wt %)		
	HDPE	LDPE	PP	PS	Liquid	Gas	Tar
min	8.000	0.000	7.290	0.000	26.330	0.000	1.000
mean	25.164	24.673	21.717	17.675	62.284	20.310	15.558
max	44.400	50.000	68.000	68.000	93.000	53.000	73.000
Std. Dev.	10.169	13.082	12.989	15.767	22.189	16.122	20.903

**Table 4:** Weights and biases used in the ANN models for the determination of: liquid, gas, and tar.

j (node)	Hidden Layer					Output Layer		
	$hw_{j1}$	$hw_{j2}$	$hw_{j3}$	$hw_{j4}$	$b_{j}$	$rw_{1j}$	$rw_{2j}$	$rw_{3j}$
1	−0.0584	−1.2180	1.6793	0.6949	−3.3497	−0.4075	−0.4075	−0.4075
2	2.4588	2.4373	−1.6909	−1.8886	−5.0976	0.4710	0.4710	0.4710
3	1.1347	−0.6826	1.2903	−3.7057	−2.9371	−2.1384	−2.1384	−2.1384
4	−2.0839	−0.7457	4.3743	−1.3009	2.7710	−2.1119	−2.1119	−2.1119
5	6.0767	0.5583	2.2405	2.1339	1.2427	2.5014	2.5014	2.5014
6	3.1857	0.5405	−0.1396	−2.4156	−3.0671	−1.8134	−1.8134	−1.8134
7	2.1866	0.4367	5.5732	0.1272	1.5037	2.4906	2.4906	2.4906
8	−2.2836	1.3779	2.9564	0.4470	1.6617	1.5278	1.5278	1.5278
9	−1.6268	3.8057	−0.1145	−0.1235	−0.3540	1.3226	1.3226	1.3226
10	−1.7640	−0.7343	1.7239	−3.4870	−0.4750	1.2533	1.2533	1.2533
11	0.3723	−3.0152	2.3341	−1.5437	1.9246	−0.7813	−0.7813	−0.7813
12	−2.0122	0.7260	−3.2506	−1.1027	−3.2196	−0.3946	−0.3946	−0.3946
13	−2.0356	−3.1544	−2.7655	0.1641	−2.1918	2.6026	2.6026	2.6026
14	−1.2101	2.1796	0.2645	0.8801	−2.7561	−1.8212	−1.8212	−1.8212
15	2.2157	−1.1864	−1.9351	1.9092	1.9727	−0.4944	−0.4944	−0.4944
					$C_{m}$	0.3628	1.0003	−1.7924
					m	**1**	**2**	**3**
